# A Novel Targeted and High‐Efficiency Nanosystem for Combinational Therapy for Alzheimer's Disease

**DOI:** 10.1002/advs.201902906

**Published:** 2020-08-09

**Authors:** Han Yang, Weihang Mu, Daohe Wei, Yue Zhang, Yue Duan, Jun‐xiao Gao, Xiao‐qun Gong, Han‐jie Wang, Xiao‐li Wu, Huaying Tao, Jin Chang

**Affiliations:** ^1^ School of Life Sciences Tianjin University 92 Weijin Road, Nankai District Tianjin 300072 P. R. China; ^2^ Department of Rehabilitation Tianjin Children's Hospital 238 Longyan Road, Beichen District Tianjin 300072 P. R. China; ^3^ Department of Neurology Tianjin Medical University General Hospital 154 Anshan Road, Heping District Tianjin 300072 P. R. China

**Keywords:** acetylcholine imbalance, Alzheimer's disease, amyloid‐beta, combinational therapy, intranasal administration

## Abstract

Alzheimer's disease (AD) remains the most prevalent neurodegenerative disease, and no effective treatment is available yet. Metal‐ion‐triggered aggregates of amyloid‐beta (A*β*) peptide and acetylcholine imbalance are reported to be possible factors in AD pathogenesis. Thus, a combination therapy that can not only inhibit and reduce A*β* aggregation but also simultaneously regulate acetylcholine imbalance that can serve as a potential treatment for AD is needed. Here, clioquinol (metal‐ion chelating agent) and donepezil (acetylcholinesterase (AChE) inhibitor) co‐encapsulated human serum albumin (HSA) nanoparticles (dcHGT NPs) are designed, which are modified with transcriptional activator protein (TAT) and monosialotetrahexosylganglioside (GM1). The GM1 lipid and TAT peptide endow this drug delivery nanosystem with high brain entry efficiency and long‐term retention capabilities through intranasal administration. It is found that dcHGT NPs can significantly inhibit and eliminate A*β* aggregation, relieve acetylcholine‐related inflammation in microglial cells, and protect primary neurons from A*β* oligomer‐induced neurotoxicity in vitro. The alleviation of A*β*‐related inflammation and AChE‐inhibited effect further synergistically adjust acetylcholine imbalance. It is further demonstrated that dcHGT NPs reduce A*β* deposition, ameliorate neuron morphological changes, rescue memory deficits, and greatly improve acetylcholine regulation ability in vivo. This multifunctional synergetic nanosystem can be a new candidate to achieve highly efficient combination therapy for AD.

## Graphical abstract

This study demonstrates that clioquinol and donepezil co‐encapsulated human serum albumin nanoparticles (dcHGT NPs) mitigate AD dysfunction to modulate A*β*‐related inflammation and neuronal damage. The alleviation of A*β*‐related inflammation and AChE inhibited effect further synergistic ally adjust acetylcholine imbalance, which inhibits the subsequent pathological cascades. dcHGT NPs could be a promising high‐efficiency targeting nanoplatform for the combination therapy of AD.

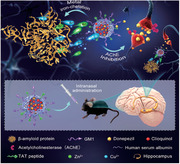

## 1 Introduction

Alzheimer's disease (AD) is a progressive age‐related chronic neurodegenerative disorder^[^ ^1^, ^2^, ^3^ ^]^ that will affect 150 million people worldwide by 2050.^[^ ^2^ ^]^ Thus, it was crucial to develop effective strategies for AD treatment. Considering the complex pathological mechanism of AD, no disease‐modifying method is currently available. The prevalent hypotheses of AD development include the amyloid hypothesis and the acetylcholine hypothesis. Aberrant aggregation of amyloid‐beta (A*β*) aberrant aggregated into amyloid fibrils and plaques, exhibited neurotoxicity, and triggered neurites injuries and proinflammatory response.^[^ ^3^, ^4^, ^5^ ^]^ Many treatment strategies are devoted to eliminating excess A*β* or suppressing A*β* plaque formation. After studies reported that metal ions could accelerate A*β* aggregation in vitro,^[^ ^6^ ^]^ the association of the disorder of metal ions in the brain with AD was further confirmed.^[^ ^7^, ^8^ ^]^ Furthermore, other research revealed that the dysfunction of acetylcholinesterase (AChE) is associated with the progression of AD.^[^ ^9^ ^]^ AChE regulates the function of the cholinergic synapse by catalyzing the hydrolysis of acetylcholine in many nervous systems diseases.^[^ ^10^, ^11^ ^]^ AChE‐induced imbalanced hydrolysis can accelerate A*β* deposition and cause inflammation‐associated cognitive decline.^[^ ^12^ ^]^ Therefore, many AChE inhibitors have been developed for AD symptomatic treatment. Given the complicated pathogenesis of AD, combination therapy offers significant therapeutic possibilities.

Clioquinol is a zinc and copper chelator that has been applied to chelate and redistribute metal‐ion‐triggered amyloid deposits both in vitro and in vivo.^[^ ^13^ ^]^ However, its short half‐life and liposoluble properties limit its application.^[^ ^13^ ^]^ Donepezil, which has potent anti‐inflammatory effects, is currently available as an acetylcholinesterase inhibitor for AD.^[^ ^14^, ^15^, ^16^ ^]^ However, its short half‐life and gastrointestinal side effects limit its availability.^[^ ^14^ ^]^ Human serum albumin (HAS) nanoparticles (NPs) have emerged as a powerful and suitable candidate for drug delivery^[^ ^1^, ^4^, ^17^ ^]^ due to their non‐immunogenicity, biodegradability, negative surface charge, and abundant modifiable groups.^[^ ^18^, ^19^, ^20^, ^21^ ^]^ Modified HSA nanoparticles are capable of sufficient brain targeting and brain accumulation. Monosialotetrahexosylganglioside (GM1), a lipid with high binding affinity to A*β*, could achieve excellent targeted effects on the AD brain.^[^ ^17^ ^]^ The transcriptional activator protein (TAT) is one of the most widely studied transmembrane peptides in the brain delivery system.^[^ ^22^, ^23^ ^]^ Thus, TAT modification could enhance the brain accumulation of nanoparticles. Furthermore, intranasal (IN) administration of nanoparticles directly introduces them into the brain via the olfactory and trigeminal nerves, which could bypass the blood–brain barrier (BBB) and avoid gastrointestinal side effects.

**Scheme 1:**
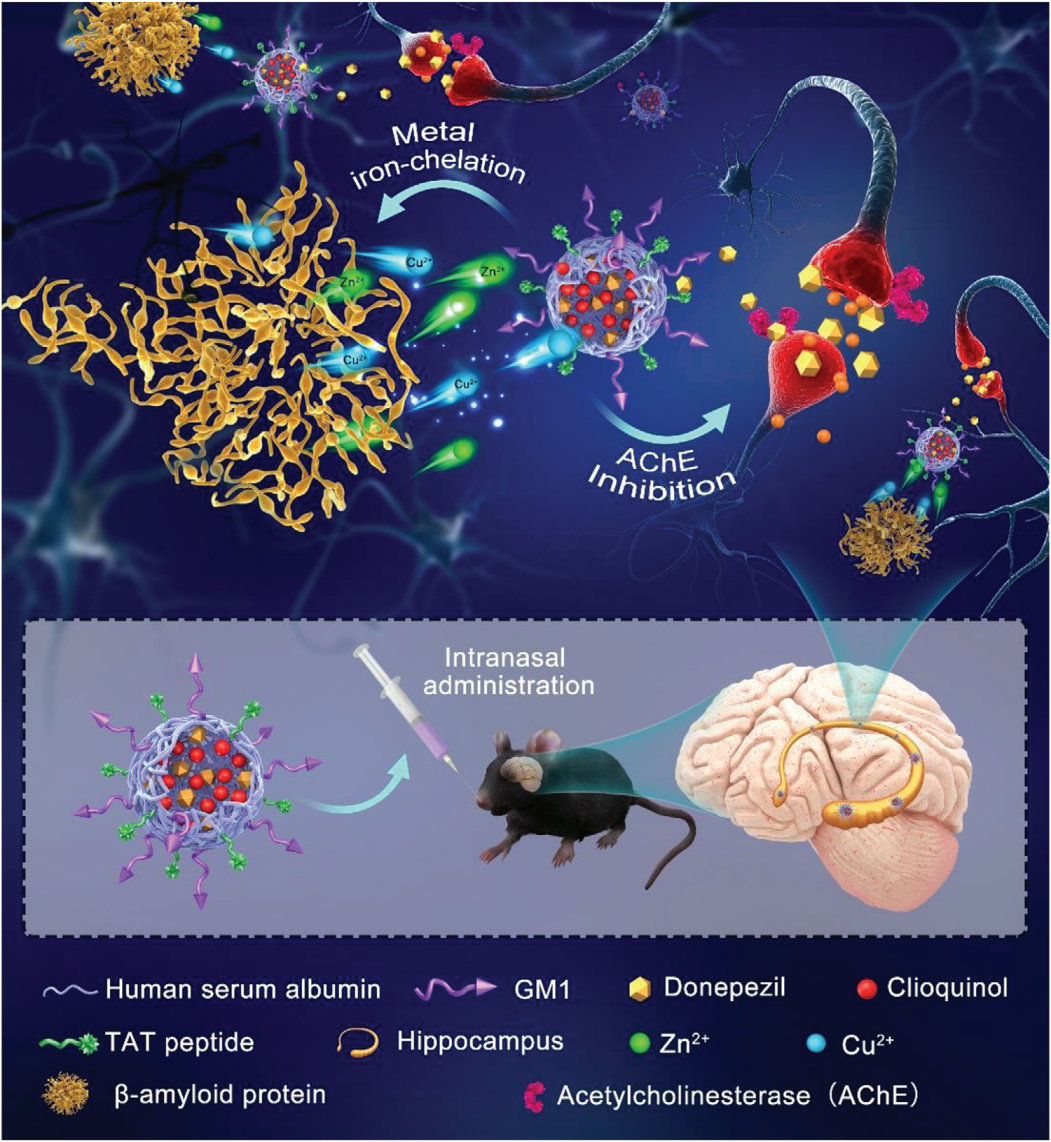
Schematic illustration of the metal iron chelation and acetylcholinesterase inhibition effects achieved by dcHGT NPs in the hippocampus after intranasal administration.

Here, integrating the metal‐ion chelation strategies against A*β* and acetylcholine imbalance, we fabricated clioquinoland donepezil co‐encapsulated human serum albumin nanoparticles (dcHGT NPs) (≈15 nm) co‐loaded with clioquinol and donepezil using the glutaraldehyde crosslink method. As illustrated in **Scheme** **1**, modification of GM1 and TAT allowed dcHGT NPs to be efficiently distributed in the hippocampus. With the slow release of clioquinol and donepezil, they displayed the functions of metal‐ion chelation and inhibition of AChE activity, respectively. Thus, the A*β* aggregation‐induced cytotoxicity and acetylcholine imbalance were remarkably alleviated, which led to neuroprotective effects and rescue of memory deficits. In this study, efficacious therapy targeting excess A*β* and acetylcholine imbalance was successfully achieved through the multifunctional dcHGT NPs in AD mice.

## 2 Results and Discussion

### 2.1 Preparation and Characterization of NPs

**Figure 1:**
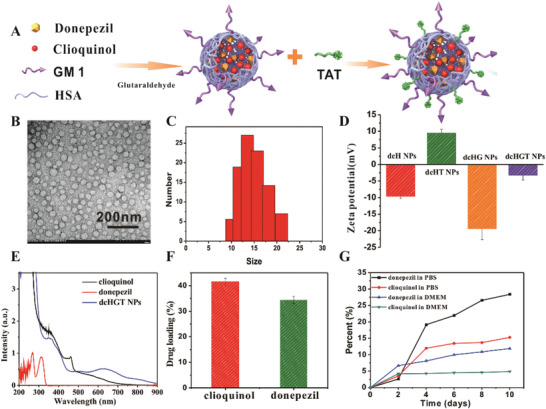
Characterization of donepezil/clioquinol @HSA‐GM1‐TAT nanoparticles (dcHGT NPs). A) Illustration of synthetic process of dcHGT NPs. B) TEM images (scale bar: 200 nm). C) DLS measurement of dcHGT NPs. D) Zeta potential of dcH NPs, dcHT NPs, dcHG NPs, and dcHGT NPs. E) UV absorption spectrum of donepezil, clioquinol, and dcHGT NPs. F) Drug‐loading efficiency of clioquinol and donepezil in dcHGT NPs. G) Drug release in PBS (pH = 7.4) and DMEM (10% FBS) as measured by UV absorption. Data are presented as the mean ± SD.

As depicted in **Figure** **1**A, dcHGT NPs were synthesized in two steps: first, due to electrostatic and hydrophobic interactions, donepezil and clioquinol were successfully encapsulated into HSA, and large amounts of GM1 were incorporated into the protein structure. Second, TAT peptides were conjugated onto the surface of donepezil/clioquinol @HSA‐GM1 (dcHG NPs). Transmission electron microscope (TEM) images indicated that both dcHG NPs (Figure S1A, Supporting Information) and dcHGT NPs (Figure 1B) had a spherical shape with a diameter of around 15 nm. The size distributions of both dcHG NPs and dcHGT NPs were measured by dynamic light scattering (DLS), which revealed average particle sizes of 13.2 and 14.6 nm, respectively (Figure S1B,C, Supporting Information). Compared with donepezil/clioquinol @HSA (dcH NPs) (−9.57 mV), the zeta potential of dcHG NPs (anionic lipid) (−19.4 mV) was more negative, and the donepezil/clioquinol @HSA‐TAT (dcHT NPs) (cationic peptide) (9.52 mV) became more positive (Figure 1D). After the successful construction of dcHGT NPs, the zeta potential was partially neutralized, which verified the efficient incorporation of GM1 and TAT. Other than the change of zeta potential, TAT was screened out by matrix‐assisted laserdesorption‐ionization time of flight (MALDI‐TOF) (Figure S1E, Supporting Information). The ultraviolet–visible (UV–vis) absorption peak (Figure 1E) and high‐performance liquid chromatography (HPLC) spectrum (Figure S1C, Supporting Information) showed the successful co‐loading of clioquinol and donepezil. Furthermore, the drug‐loading efficiency of clioquinol and donepezil reached 41% and 35%, respectively (Figure 1F; Figure S1D, Supporting Information). The release of two drugs was examined in phosphate buffer saline (PBS) (pH = 7.4) and Dulbecco's modified eagle medium (DMEM). dcHGT NPs presented a slow release of clioquinol and donepezil, which could reach 27% and 15%, respectively, in 10 days. Moreover, dcHGT NPs presented more potential in steady and long‐term drug release of clioquinol (5.3%) and donepezil (10%) in DMEM (10% fetal bovine serum (FBS)) than PBS (pH = 7.4) (Figure 1G). Taken together, these findings indicated that clioquinol and donepezil co‐loaded HSA nanoparticles incorporating TAT and GM1 were successfully constructed.

**Figure 2:**
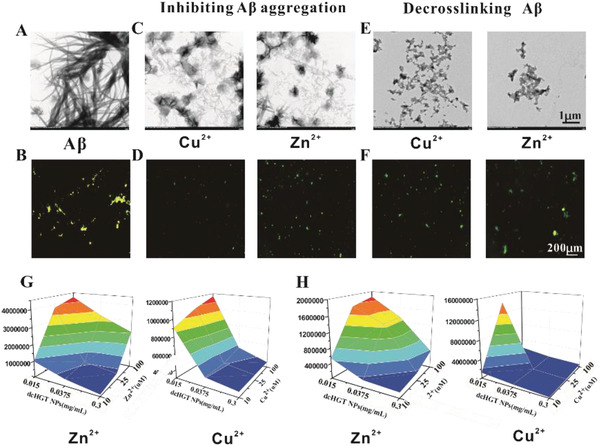
A*β* aggregation inhibition and A*β* fibril de‐crosslinking effects of dcHGT NPs. A) TEM and B) ThT fluorescence images of A*β* fibrils. Inhibiting A*β* aggregation: C) TEM and D) ThT fluorescence images of A*β* monomers incubated with dcHGT NPs and metal ions. Disaggregation experiments: E) TEM and F) ThT fluorescence images of A*β* fibrils incubated with dcHGT NPs and metal ions (A*β* = 100 × 10^−6^ m, metal = 25 × 10^−6^ m, and dcHGT NPs = 0.15 mg mL^−1^). 3D color map surface of fluorescence signals of G) inhibition and H) disaggregation experiments (A*β* = 10 × 10^−6^ m, metal ions = 10–100 × 10^−6^ m, and dcHGT NPs = 0.015–0.15 mg mL^−1^). Data are presented as the mean ± SD.

### 2.2 Inhibition and Disaggregation Effects toward A*β*1‐42 of the NPs

To investigate the effect of dcHGT NPs on inhibiting A*β* aggregation and facilitating A*β* disaggregation, the aggregation kinetics of A*β* fibrils were observed and measured via TEM and thioflavin T (ThT) assay. The A*β*1‐42 treated with metal ions was incubated to form aggregate fibrils and imaged with TEM (**Figure** **2**A), showing rapidly increased ThT fluorescence intensity (FI) (Figure 2B). For inhibition experiments, fresh A*β*1‐42 was incubated with metal ions and dcHGT NPs. The morphology of these few and amorphous A*β* fibrils was broadly observed with TEM (Figure 2C), indicating the inhibition of amyloid formation. In disaggregation experiments, dcHGT NPs were added to A*β* fibrils to verify their de‐crosslinking effects, and their distinct structural variation was visible under TEM (Figure 2E). The change in ThT fluorescence intensity was in accord with the TEM results. Under incubation with dcHGT NPs, the ThT fluorescence intensity was remarkably decreased (Figure 2D–F). Interestingly, dcHGT NPs revealed stronger inhibition and disaggregation effects toward Cu‐triggered A*β*1‐42 aggregation. This result was consistent with previous studies indicating that A*β*1‐42 has a higher affinity for Cu^2+^.^[^ ^8^ ^]^ We further measured the ThT fluorescence variation at concentrations of A*β* ranging from 10 × 10^−3^ to 100 × 10^−3^ m, dcHGT NPs ranging from 0.015 to 0.3 mg mL^−1^, and metal ions ranging from 10 × 10^−3^ to 100 × 10^−3^ m. The ThT fluorescence variation trends are presented in a 3D color map. The ThT fluorescence increased with increasing ion concentration and decreased with increased dcHGT NPs’ concentration (Figure 2G,H; Figure S2, Supporting Information). All of these results indicated that dcHGT NPs exhibited a concentration‐dependent A*β* fibrils inhibition and disaggregation effects in vitro, with particularly high effectiveness for Cu‐triggered A*β* fibrils.

### 2.3 Biocompatibility, Cellular Uptake, Degradation, and Inflammatory Inhibition of the NPs

**Figure 3:**
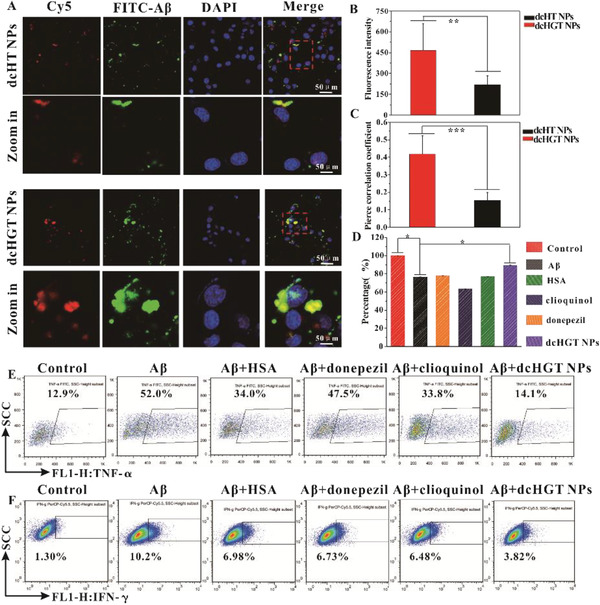
Effects of dcHGT NPs on cellular uptake, distribution, and inflammatory mitigation. A) Cellular uptake of FAM‐A*β*1‐42 with Cy5‐dcHT NPs/Cy5‐dcHGT NPs. Scale bar: 50 µm. B) Statistical analysis of the cellular uptake of FAM‐A*β*1‐42 and Cy5‐dcHT/Cy5‐dcHGT NPs. C) Statistical analysis of co‐localization between FAM‐A*β*1‐42 and Cy5‐dcHT/Cy5‐dcHGT NPs. D) Mitigation of A*β*‐mediated inflammation of HSA, donepezil, clioquinol, and dcHGT NP groups. Flow cytometry of the expression of E) TNF‐*α* and F) IFN‐*γ* in BV‐2 cells collected in an inflammatory mitigation experiment. Data are presented as the mean ± SD. *P < 0.05, **P < 0.01, and ***P < 0.001.

BV‐2 cells were cultured with dcHGT NPs, HSA, donepezil, and clioquinol to evaluate their biocompatibility. 3‐(4,5‐dimethyl‐2‐thiazolyl)‐2,5‐diphenyl‐2‐H‐tetrazolium (MTT) bromide results showed cytotoxicity of clioquinol and donepezil to BV‐2 cells at different concentrations. However, HSA and dcHGT NPs exhibited better biocompatibility compared with clioquinol and donepezil (Figure S3, Supporting Information). To further assess the A*β*‐binding affinity, the nanocomposites were labeled with Cyanine5 (Cy5) for cellular study. Microglia are crucial phagocytes that mediate intracerebral cellular A*β* clearance.^[^ ^24^ ^]^ Therefore, to verify the targeting effect of dcHGT NPs toward A*β*1‐42, BV‐2 cells were incubated with Carboxyfluorescein (FAM)‐labeled A*β*1‐42 and dcHGT NPs. As shown in **Figure** **3**A, the BV‐2 cells exhibited strong red fluorescence signals, which indicated that the dcHT NPs and dcHGT NPs were taken up by BV‐2 cells efficiently. Interestingly, the cells treated with dcHGT NPs exhibited 2.15 times stronger red fluorescence signals than dcHT NPs (Figure 3B), suggesting that more dcHGT NPs were endocytosed by BV‐2 cells. The co‐localization correlations between the green fluorescence signals emitted by A*β*1‐42 and the red fluorescence signals emitted by dcHT NPs or dcHGT NPs were further measured and analyzed. Statistical analysis showed that the co‐localization correlation of dcHGT NPs with A*β*1‐42 was 2.7 times higher than that of dcHT NPs. Due to the GM1 affinity to A*β*1‐42, more dcHGT NPs were co‐localized with A*β*1‐42 (Figure 3C).

To further confirm the endocytic pathway, Fluorescein isothiocyanate isomer (FITC)‐labeled dextran, FITC‐labeled cholera toxin subunit B, and FITC‐labeled transferrin were chosen as macro‐pinocytosis, caveolae, and clathrin pathway markers, respectively. Compared with transferrin and dextran, dcHGT NPs and cholera toxin subunit B presented a significant co‐localization in which the Pearson's correlation coefficient (PCC) was ≈0.48 (Figure S4, Supporting Information). This result indicated that the uptake of dcHGT NPs mainly took the advantage of the caveolae pathway.

Moreover, the co‐localization between dcHGT NPs and lysosome was imaged, and PCC between them in BV‐2 cells was ≈0.74 (Figure S5, Supporting Information). This result suggested that, after being endocytosed into the cell, most dcHGT NPs were transported to lysosomes for degradation.

Additionally, the onset of AD is often accompanied by inflammation in the brain.^[^ ^25^ ^]^ The regulation of acetylcholine imbalance could intercede peripheral and central anti‐inflammatory responses. By activating the cholinergic anti‐inflammatory pathway (CAIP), the release of inflammatory factors such as Tumor Necrosis Factor‐alpha (TNF‐*α*) is decreased.^[^ ^12^ ^]^ An oligomeric A*β*‐mediated microglia inflammation model^[^ ^26^, ^27^ ^]^ was established to analyze the regulatory function of dcHT NPs toward acetylcholine imbalance. After treatment with A*β* oligomers, cell viability in HSA, donepezil, clioquinol, and dcHGT NP groups was measured. As expected, the dcHGT NP group showed remarkably higher viability (89%) than the HSA (77%), donepezil (78%), and clioquinol (63%) groups compared with the A*β* (76%) group (Figure 3D), indicating the excellent protective effect of dcHGT NPs. Meanwhile, as shown in Figure 3E,F, A*β* oligomers caused a rapid increase of TNF‐*α* (52.0%) and interferon‐gamma (IFN‐*γ*) (10.2%) compared with the control group, suggesting that the A*β* oligomers induced inflammation. Furthermore, compared with the HSA, donepezil, and clioquinol groups, the dcHGT NP group showed the greatest reduction of TNF‐*α* (14.1%) and IFN‐*γ* (3.82%). The drastic decrease of inflammatory factors (TNF‐*α* and IFN‐*γ*) in the dcHGT NP group indicated the best anti‐inflammatory response. Notably, these findings suggest that dcHGT NPs play an essential role in the regulation of acetylcholine imbalance.

### 2.4 NPs Protect Neurons from A*β*1‐42 Oligomer‐Induced Toxicity

**Figure 4:**
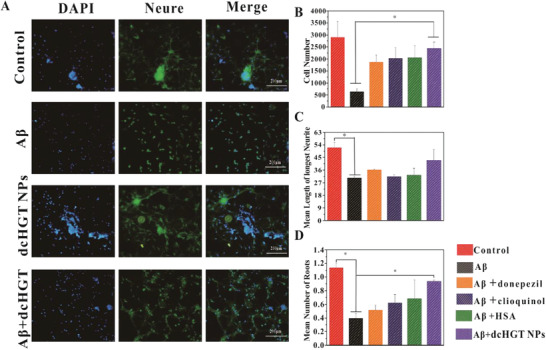
Neuroprotection of dcHGT NPs in A*β* oligomer‐induced neuronal damage. A) Fluorescent morphology images of the primary neurons in control, A*β*, dcHGT NPs, and A*β*+dcHGT NP groups captured by HCS (scale bar: 200 µm). Statistical analysis of B) the neuronal cell number, C) the mean length of the longest neurite, and D) the mean number of roots. Data are presented as the mean ± SD. *P < 0.05.

Previous studies reported that A*β* oligomers cause the destruction of synapses and neuron apoptosis, which results in memory impairment, and the health of neurons is an essential indicator of the effectiveness of AD treatment.^[^ ^28^, ^29^, ^30^ ^]^ Furthermore, synaptic damage occurs long before neuron apoptosis;^[^ ^31^ ^]^ thus, the value changes in synapses are an important manifestation of neuronal damage. Here, we investigated the treatment capacity of donepezil, clioquinol, HSA, GM1, TAT, and dcHGT NPs for A*β* oligomer‐mediated neuronal damage. As shown in **Figure** **4**A, the toxicity of A*β* oligomers caused significant damage to cell viability, major loss of roots, and neuron length injury. Donepezil, clioquinol, HSA, GM1, and dcHGT NPs showed different degrees of mitigation of A*β* oligomer‐induced neuronal damage. The donepezil, clioquinol, GM1, and HSA groups only showed effective protection from neurons’ apoptosis, with negligible protection of neurite length or root number. Interestingly, dcHGT NPs displayed the best neuroprotective effect. The neurites were richly reticulated and roots were well preserved, with little neurons apoptosis (Figure 4A; Figure S6, Supporting Information). Statistical analysis further revealed that the number of neuronal cells was increased by 227% in the dcHGT NP group compared with the A*β* oligomer group (Figure 4B). Moreover, the dcHGT NP group showed better protective effects than the donepezil, clioquinol, and HSA groups in the length and root number of the neurons (Figure 4C,D). The expression of apoptosis‐related protein caspase 3 is usually used to indicate apoptosis, which can be used to measure nerve damage.^[^ ^32^ ^]^ In addition to being involved in the brain's learning and memory functions, neurotype 7 nicotine receptors *α*7nAChR (CHRNA7) have an obvious neuroprotective effect.^[^ ^33^ ^]^ What is more, synaptophsin (SYAP1)^[^ ^34^ ^]^ and growth‐related protein 43 (GAP43)^[^ ^35^ ^]^ have been identified as synaptic remodeling proteins. Immunofluorescence staining observations of synaptophysin (SYAP1) and growth associated protein‐43 (GAP‐43) in dcHGT NP group exhibited a dramatic increment of PC12 (Figures S7A and S8A, Supporting Information). The following flow quantitative analysis conformed the change trend of immunofluorescence (Figures S7B,C and S8B,C, Supporting Information). Collectively, these results indicate that dcHGT NPs relieve the neurite and root damage and neuron apoptosis induced by A*β* oligomer‐mediated neuronal toxicity.

To further confirm the neuroprotection of the dcHGT NPs, the expression level of the neuroprotective receptor CHRNA7 and neuronal cell apoptosis‐related proteins caspase 3 was quantified by flow cytometry and enzyme linked immunosorbent assay (ELISA) (Figure S9, Supporting Information). It was found that CHRNA7 was upregulated in donepezil, clioquinol, dcHGT NP groups, especially in dcHGT NP. Compared with other groups, caspase 3 production was observed to be significantly downregulated in dcHGT NP confirmed that dcHGT NPs showed much stronger neuroprotection and downregulation of the A*β* oligomer‐induced neurons’ apoptosis.

### 2.5 Biodistribution and Brain Accumulation of NPs In Vivo

**Figure 5:**
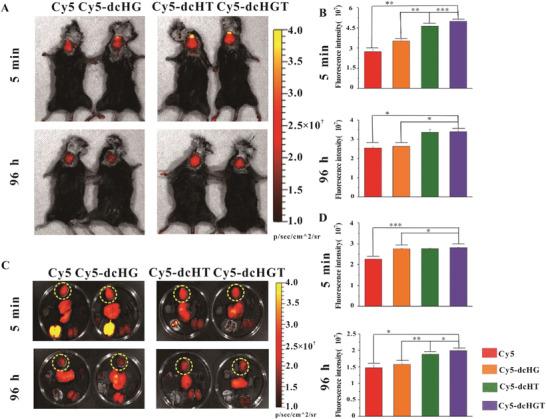
In vivo imaging of Cy5‐dcHGT NPs in AD mice. A) In vivo fluorescence imaging and B) quantitative fluorescence analysis at 5 min and 96 h after nasal administration of Cy5, Cy5‐dcHG NPs, Cy5‐dcHT NPs, and Cy5‐dcHGT NPs (*n* = 3 per group). C) Ex vivo fluorescence imaging and D) quantitative fluorescence analysis at 5 min and 96 h after administration (brains are marked with a yellow dotted circle). Data are presented as the mean ± SD. *P < 0.05, **P < 0.01, and ***P < 0.001.

Given the transmembrane and A*β* affinity effects of the dcHGT NPs, we investigated their biodistribution and brain accumulation in vivo. Compared with the Cy5‐free group, the fluorescence intensity of Cy5‐dcHG NPs, Cy5‐dcHT NPs, and Cy5‐dcHGT NPs was significantly increased after 5 min and showed mass retention in the brain after 96 h (**Figure** **5**A; Figure S10A, Supporting Information). Notably, the fluorescence intensity of the Cy5‐dcHGT NP group was 1.9 times higher than that of the Cy5‐free group after 5 min and maintained that advantage at 96 h (Figure 5B). The mice were sacrificed, and the fluorescence intensity of the organs and brain was further examined. Modification with TAT enhanced Cy5‐dcHT NP and Cy5‐dcHGT NP accumulation in the brain but not in the lung at 5 min, indicating the transmembrane ability of TAT. Cy5‐dcHGT NPs showed retentional advantages in the brain after 96 h (Figure 5C; Figure S10B, Supporting Information). These results were further confirmed by statistical analysis (Figure 5D). The gathering of Cy5‐dcHGT NPs was visible after 96 h in the hippocampal region of the brain (Figure S11, Supporting Information). Above all, our results confirmed that Cy5‐dcHGT NPs modified with TAT and GM1 achieved significant accumulation in the brain in a short period and showed prolonged brain retention.

### 2.6 NPs Reduce A*β* Deposition, Alleviate Acetylcholine Imbalance, Ameliorate Neurologic Damage, and Rescue Memory Deficits in AD Model Mice

**Figure 6:**
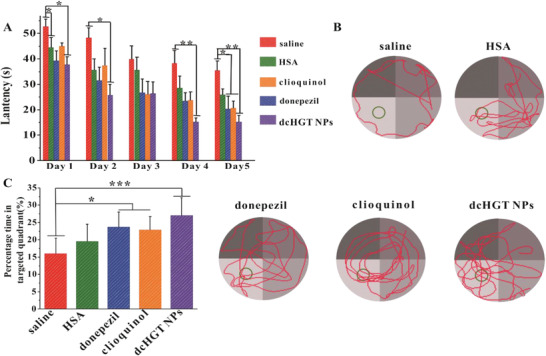
Neuroprotection and memory deficit rescue of dcHGT NPs in AD mice. A) Escape latency of saline, donepezil, clioquinol, HSA, and dcHGT NP groups (*n* = 8 per group). B) Representative swimming paths of the five groups. C) Percentage of the time spent in the target quadrant in the five groups. Data are presented as the mean ± SD. *P < 0.05, **P < 0.01, and ***P < 0.001.

Given the in vitro therapeutic effects of dcHGT NPs, we further explored their impacts on neuroprotection and rescuing of memory deficits in β‐amyloid precursor protein/presenilin‐1 (APP/PS1) AD mouse models. The AD mice were randomly divided into five treatment groups: saline, donepezil, clioquinol, HSA, and dcHGT NPs. The spatial learning and memory of the mice were assessed via the Morris water maze (MWM) test. As shown in **Figure** **6**A, during the 5 day training session, the donepezil, clioquinol, and HSA groups showed a slight improvement in the learning performance of escape latency. Notably, the dcHGT NP group exhibited significant improvement in learning performance compared with the saline group (*P* < 0.01). In the space exploration experiment, the trajectory and percentage of time spent exploring in the target quadrant of each group were recorded and analyzed. Compared with the donepezil, clioquinol, and HSA groups, the mice in the dcHGT NP group spent the longest time exploring the target platform quadrant. Remarkably, the percentage of exploration time in the target platform quadrant of the dcHGT NP group was 68.9% higher than that of the saline group (Figure 6B,C). Our findings demonstrated that the dcHGT NPs were able to achieve significant improvement in learning and space exploration ability.

**Figure 7:**
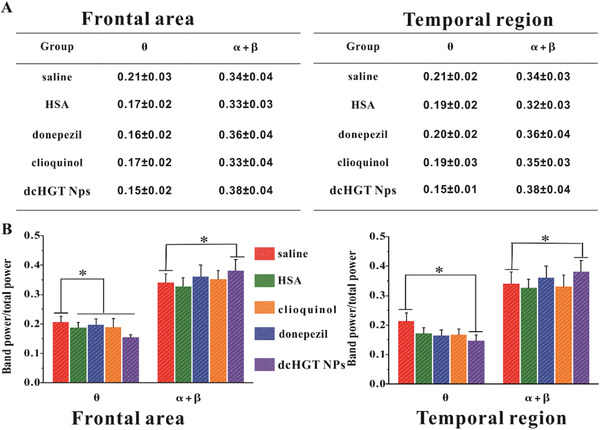
Neuroprotection and acetylcholine imbalance alleviation of dcHGT NPs. A) Percentage of high‐frequency *α* and *β* wave and low‐frequency *θ* wave power in frontal and temporal regions of the saline, donepezil, clioquinol, HSA, and dcHGT NP groups (*n* = 8 per group). B) Statistical analysis of band power/total power in frontal and temporal regions in five groups. Data are presented as the mean ± SD. *P < 0.05.

An electroencephalogram (EEG) is the sum of postsynaptic potentials in the cerebral cortex, which can reflect the overall functional state of the electrophysiological activity of the cranial nervous system with high sensitivity.^[^ ^36^, ^37^, ^38^ ^]^ Degenerative cholinergic neurons and decreased acetylcholine content in the synaptic cleft have been shown to lead to changes in synaptic potential.^[^ ^36^, ^37^, ^39^ ^]^ Synaptic potential synchronization disorder presents as an increase of low‐frequency *θ* waves. EEG usually shows high‐frequency *α* wave and *β* wave activity reduction and a low‐frequency *θ* wave activity increase in AD patients.^[^ ^40^, ^41^, ^42^ ^]^ We assessed the frequency segments of frontal and temporal regions in saline, donepezil, clioquinol, HAS, and dcHGT NP groups to detect the acetylcholine levels (**Figure** **7**A). As shown in Figure 7B, the donepezil, clioquinol, HSA, and dcHGT NP groups exhibited a significant decrease in low‐frequency *θ* wave power value in the frontal area. However, in the temporal regions, only the dcHGT NP group showed a significantly decreased *θ* wave power value compared with the saline group (*P* < 0.05). Similarly, dcHGT NPs presented a high‐frequency wave (*α* + *β*) enhancement in the frontal and temporal areas (Figure 7B) (*P* < 0.05). The preferable electrophysiological activity function of the dcHGT NP group illustrated the excellent acetylcholine protective effects in AD mice.

**Figure 8:**
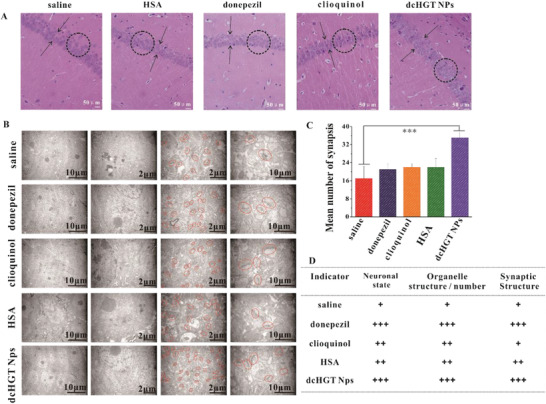
Morphological studies of dcHGT NPs’ neuroprotective effects. A) H&E staining of the dentate gyrus region in saline, donepezil, clioquinol, HSA, and dcHGT NP groups. The cell layers are indicated by arrowheads, and the circles contain the characteristic arrangement of cells. Scale bar: 50 µm. B) TEM observation of hippocampal ultrastructure in the five groups. C) Quantitative analysis of synapse number in five groups. D) Evaluation of the nerve cells’ overall morphology and activity in the hippocampus of the five groups. Data are presented as the mean ± SD.***P < 0.001.

Finally, the neuroprotective performance of dcHGT NPs was morphologically studied using hematoxylin and eosin (H&E) staining and TEM imaging. As shown in **Figure** **8**A, unconsolidated cell arrangement and reduced cell layers, and cell numbers were observed in the saline group. In the donepezil, clioquinol, and HSA groups, the unconsolidated arrangement of cells significantly improved. Remarkably, only the dcHGT NP group showed obvious amelioration of the decreases in cell layers and cell numbers (Figure 8A).

To further investigate the neuroprotective effects of dcHGT NPs, the nerve cell population, the number and structure of organelles in the nerve cells, and the structure and activity of synapses were examined. As shown in Figure 8B, the dcHGT NP group had a higher organelle abundance and synapse number than the other groups. Furthermore, in the dcHGT NP group, the synaptic structure was more legible, and presynaptic dilatation was dense with more vesicles, which suggested that the neurons were healthy and vibrant. Statistical data indicated that the synapse number of the dcHGT NP group was 2.2 times higher than that of the saline group (*P* < 0.001; Figure 8C). The data on the nerve cell population, the number and structure of organelles in nerve cells, and the structure and activity of the synapses are shown in Figure 8D.

**Figure 9:**
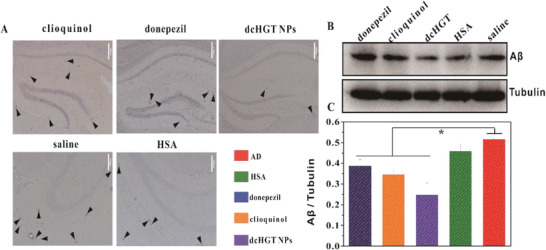
dcHGT NPs reduced A*β* deposition. A) Immuno‐histochemical staining of A*β* deposition in brain sections of the saline, donepezil, clioquinol, HSA, and dcHGT NP groups. Scale bar: 200 µm. Brown spots are pointed out by arrowheads in the hippocampus of AD mice. B) Western blot analysis and C) quantification of A*β* levels in five groups, with tubulin as a loading control. Data are presented as the mean ± SD. *P < 0.05.

To evaluate the ability of dcHGT NPs to decrease A*β* deposition, immuno‐histochemical analysis and immunoblot analysis were performed. The donepezil, clioquinol, and HSA groups showed a negligible A*β* deposition decrease, but the dcHGT NP group showed a significant decline of A*β* deposition (**Figure** **9**A). Immunoblot analysis further confirmed the significantly decrease of A*β* content in the dcHGT NP group (Figure 9B,C). In general, dcHGT NPs effectively decreased A*β* deposition and moderated nerve injury.

## 3 Conclusion

In this study, we construct a multifunctional HSA nanosystem (dcHGT NPs), which could mitigate A*β*‐mediated neurotoxicity and regulate acetylcholine imbalance in AD mice. After incorporating GM1 and TAT into HSA nanoparticles, dcHGT NPs exhibited much higher A*β*1‐42 binding affinity and transmembrane efficiency, which could increase brain accumulation with excellent biocompatibility. Moreover, the results indicated dcHGT NPs inhibited A*β* aggregation and facilitated A*β* disaggregation through chelation of metal ion (Cu^2+^ and Zn^2+^), with particularly high effectiveness for Cu‐triggered A*β* fibrils. Taken advantage of caveolae pathway, dcHGT NP was enriched in microglial cell and then transported to the lysosome for degradation. Compared with HSA, donepezil, clioquinol groups, the dcHGT NP group significantly switch the A*β* oligomer‐induced inflammation in microglia, which gives rise to a drastic decrease of the proinflammatory cytokines (TNF‐*α* and IFN‐*γ*). dcHGT NP also upregulated the expression of neuroprotective proteins (CHRNA7, SYAP1, and GAP43) and downregulated apoptosis‐related protein caspase 3 simultaneously. What is more, dcHGT NPs achieve protection of neuron morphology in A*β* oligomer‐mediated neuronal damage. After 1 month intranasal administration of dcHGT NPs, it largely rescued spatial learning and memory deficit, ameliorated acetylcholine imbalance, decreased A*β* deposition, and moderated the neuronal damage of APP/PS1 mice.

In summary, this study demonstrated that the dcHGT NPs mitigated the AD dysfunction via two collaborative progressive methods: 1) inhibiting A*β* aggregation and facilitating A*β* disaggregation to modulate A*β*‐related inflammation and neuronal damage and 2) the alleviation of A*β*‐related inflammation and AChE‐inhibited effect further synergistically adjusted acetylcholine imbalance, which inhibit the subsequent pathological cascades as a promising high‐efficiency nanoplatform for the combination therapy of AD. We believe that such a high‐efficiency nanosystem can pave the way for efficient combination therapy for neurodegenerative disorders in the future.

## 4 Experimental Section

##### Methods—Materials

Donepezil, clioquinol, HSA, ThT, 1‐(3‐dimethylaminopropyl)‐3‐ethylcarbodiimide hydrochloride (EDC), *N*‐hydroxysuccinimide (1‐hydroxypyrrolidine‐2,5‐dione, NHS), and FITC‐dextran were purchased from Sigma–Aldrich; GM1 was purchased from J&K(Beijing); TAT peptide and A*β*1‐42 were purchased from GL Biochem (Shanghai), and the purity of the peptides was greater than 98%; glutaric dialdehyde was purchased from Aladdin (Shanghai); hexafluoroisopropanol (HFIP) and 4‐(*N*‐maleimidomethyl) cyclohexane‐1‐carboxylic acid 3‐sulfo‐*N*‐hydroxysuccinimide ester sodium salt (Sulfo‐SMCC) were purchased from Yuanye (Shanghai). FITC‐transferrin, FITC‐cholera toxin subunit B, and LysoTracker Red probe were purchased from Invitrogen (USA). Milli‐Q water (18 MΩ cm) was used in the experiments. Nicotinic acetylcholine receptors *α*7(*α*7 nAChR), SYAP1 and GAP43 antibody were purchased from Bioss (Beijing). Rat cysteine protease‐3 ELISA kit was purchased from Huyu Biotechnology (Shanghai).

##### Cells

Murine microglial BV‐2 cells were maintained in DMEM containing 10% inactivated FBS, 1% penicillin/streptomycin under 37 °C and 5% CO_2_. High differentiation PC12 were maintained in Roswell Park Memorial Institute (RPMI) 1640 medium containing 10% inactivated FBS, 1% penicillin/streptomycin under 37 °C and 5% CO_2_. Primary hippocampal neurons cells were derived from the hippocampus of fetal rats gained from Sprague–Dawley (SD) rats after 16–18 days of pregnancy. Briefly, the embryos and the brains were collected and rinsed by ice‐cold PBS. The hippocampus tissues were dissected and transferred to papain enzyme for 21 min trypsinization. The trypsinization was ended by adding DMEM containing 20% FBS (v/v), 1% penicillin/streptomycin, and 0.5 × 10^−3^ m glutamax. After centrifuged at 1400 rpm for 7 min, cells were resuspended in neurobasal medium supplemented with 2% B27 Plus Supplement, 1% penicillin/streptomycin, and 0.5 × 10^−3^ m glutamax. Then the cells were seeded on poly‐l‐lysine (0.1 mg mL^−1^, Sigma)‐coated 24‐well plates at 2 × 10^5^ cells per well.

##### Animals

SD rats and 6 month male APP/PS1 transgenic mice were obtained from Shandong Creation Bioscience Co, INC, and housed protocol was approved by the Animal Ethics Committee of Institute of Radiation, Chinese Academy of Medical Sciences.

##### Synthesis and Characterization of dcHG NPs and dcHGT NPs

A total of 25 µL of donepezil (100 mg mL^−1^, in dimethyl sulfoxide (DMSO)), 35 µL of clioquinol (50 mg mL^−1^, in DMSO), and 10 µL GM1 (100 mg mL^−1^) of aqueous solution were mixed together and added into 2 mL of aqueous solution of HAS (100 mg mL^−1^) under stirring. The reaction solution was made with the addition of 70 µL of glutaraldehyde solution and stirred for 3 h. dcHG NPs were obtained by centrifuging and purifying three times by a Milli‐Q water purification system with a 100 kDa ultrafiltration tube at 4000 rpm. dcHG NPs were modified and TAT was obtained via amidation. The TAT aqueous solution was added to HSA–GM1 at a molar mass ratio of 1:5 (TAT:HSA) after the carboxyl groups on the surface of dcHG NPs were activated by EDC/NHS and modified by Sulfo‐SMCC. After incubating with stirring overnight, the free TAT was removed and purified with centrifugation as above.

The size and zeta potential were characterized by the Zetasizer Nano series (Malvern Instruments); TEM images were taken by a JEOL TEM 2010F transmission electron microscope at an operating voltage of 200 kV in bright‐field mode after negative staining with a 0.3% uranyl acetate solution; an HPLC system (Agilent, USA) was used to acquire the UV spectrum. Mass spectrometry was characterized by a MALDI‐TOF (Bruker Daltonics, USA).

##### Drug Loading Efficiency and Drug Release of dcHGT NPs

For analysis purposes, the dcHGT NPs were sonicated by an ultrasonic disrupter for 5 min and dissolved in DMSO. Then the mixed solution was measured by an HPLC system (Agilent). The drug‐loading efficiency was calculated when the absorbance readings of donepezil, clioquinol, and HSA at 265 nm were determined. The drug release experiment was carried out within 10 days in PBS (pH = 7.4) and DMEM separately. The sample was measured every 2 days as described above.

##### Preparation of A*β*1‐42 Oligomers and Fibrils

A*β*1‐42 oligomers and fibrils were prepared as previously described. Briefly, A*β*1‐42 was dissolved and stored in HFIP before use. After evaporating the HFIP, the peptide was redissolved in DMSO for sonication. Subsequently, the above DMSO solution of A*β*1‐42 was used for the preparation of A*β*1‐42 oligomers. A*β*1‐42 oligomers were obtained by diluting the solution with deionized water to 100 × 10^−6^ m and incubated at 4 °C for 24 h. The A*β*1‐42 fibrils were induced by adding Zn^2+^ or Cu^2+^ metal ions into the peptide after 24 h of co‐incubation at 37 °C.

##### A*β* Fibril Inhibition and Disaggregation Experiments

To study the effect of dcHGT NPs on metal‐induced A*β* fibrils, inhibition and disaggregation experiments were performed. In the inhibition study, 10 × 10^−6^, 25 × 10^−6^, and 100 × 10^−6^ m A*β*1‐42 peptide were treated with 10 × 10^−6^, 25 × 10^−6^, and 100 × 10^−6^ m Zn^2+^ or Cu^2+^ metal ions for 2 min at room temperature. Then 20 µL of dcHGT NPs (0.015, 0.0375, and 0.3 mg mL^−1^) was introduced for 24 h at 37 °C. Similarly, a disaggregation experiment was performed by adding 20 µL of dcHGT NPs (0.015, 0.0375, and 0.3 mg mL^−1^) into 10 × 10^−6^, 25 × 10^−6^, and 100 × 10^−6^ m A*β*1‐42 fibrils for 24 h at 37 °C. The effects of A*β* fibril inhibition and disaggregation were confirmed by the TEM images and the ThT fluorescence assay. In order to trace the aggregation of A*β*1‐42, ThT (10 × 10^−6^ m) was mixed into the solution and incubated for 1 h. ThT fluorescence images were acquired using an inverted Olympus fluorescence microscope (IX‐51).

##### Internalized and Fluorescence Co‐Localization Assay

To capture the internalization and co‐localization of Cy5‐dcHGT NPs between FAM‐A*β*1‐42 in BV‐2 cells, 1 × 10^5^ BV‐2 cells were seeded and cultivated in a confocal dish overnight. Then the cells were co‐cultured with 2 µg mL^−1^ FAM‐A*β*1‐42 and Cy5‐dcHT NPs/Cy5‐dcHGT NPs (0.01 mg mL^−1^). And 4 h later, cells were fixed and stained by 4',6‐diamidino‐2‐phenylindole (DAPI) (100 ng mL^−1^) for 5 min and imaged by an inverted Olympus fluorescence microscope (IX‐51). Cellular co‐localization of FAM‐A*β*1‐42 and Cy5‐dcHT NPs/Cy5‐dcHGT NPs was analyzed by ImageJ software.

##### Cellular Internalization Pathways and Degradation of Cy5‐dcHGT NPs

Around 1 × 10^5^ BV‐2 cells were seeded in a confocal dish and cultured overnight. The cells were co‐cultured with Cy5‐dcHGT NPs (0.01 mg mL^−1^) in the presence of endocytosis marker of FITC‐transferrin (clathrin‐mediated endocytosis, 0.1 mg mL^−1^), FITC‐dextran (micro‐pinocytosis‐mediated, 0.2 mg mL^−1^), or FITC‐cholera toxin subunit B (caveolae‐mediated, 5 µg mL^−1^) for 2 h. Then cells were washed and stained with Hoechst 33342(10 µg mL^−1^) for 10 min and imaged by an inverted Olympus fluorescence microscope (IX‐51). The overlap ratio *R* was analyzed by Image J.

Then, around 1 × 10^5^ BV‐2 cells were seeded in confocal dish and cultured overnight, and then incubated with Cy5‐dcHGT NPs (0.01 mg mL^−1^) for 4 h, respectively. To visualized the endosomes/lysosomes and nucleus, BV‐2 cells were labeled with 50 × 10^−9^ m LysoTracker Red probe for 30 min and Hoechst 33342 (10 µg mL^−1^) for 10 min. Thereafter, BV‐2 cells were imaged by an inverted Olympus fluorescence microscope (IX‐51) and analyzed by Image J.

##### Flow Cytometry Analysis

BV‐2 cells were seeded for 24 h in a 24‐well plate (1 × 10^5^ cells per well) before nanoparticle incubation. Cells were treated with donepezil (0.05 mg mL^−1^), clioquinol (0.03 mg mL^−1^), HSA (0.01 mg mL^−1^), and dcHGT NPs (0.01 mg mL^−1^) under the presence of 20 × 10^−6^ m A*β*1‐42 oligomers in fresh culture medium. After incubation for 24 h, BV‐2 cells were washed and fixed by 4% paraformaldehyde. After staining for 30 min, cells were analyzed by flow cytometry.

##### Neurite Outgrowth Assay

Primary neurons were seeded in 24‐well plates (2 × 10^5^ per well) and cultured for 7 days. Then the medium was replaced with medium containing HSA, GM1, donepezil, clioquinol, TAT, and dcHGT NPs in the presence of 20 × 10^−6^ m A*β*1‐42 oligomers. After incubated for 48 h at 37 °C, neurons were fixed and blocked (5% goat serum) before performing Microtubule‐associated protein‐2 (MAP2) (1:50) immunofluorescent staining. FITC‐conjugated secondary antibodies (1:50) were incubated to visualize neurons and DAPI was used for nuclear location. Cells were imaged using the HCS instrument, and the number of neurite, mean length of longest neurite, and mean number of roots were analyzed via the Thermo Scientific Cellomics Neuronal Profiling Bioapplication software.

##### Fluorescence Imaging of Cy5‐dcHGT NPs In Vivo

About 30 µL of Cy5‐dcHGT NPs, Cy5‐dcHG NPs, Cy5‐dcHT NPs (at an HSA dose of 10 mg kg^−1^), and Cy5 solution (5 mg kg^−1^) was nasally administrated into APP/PS1 transgenic mice. An ex vivo/in vivo imaging system (Maestro, USA) was used for in vivo fluorescence imaging at 5 min, 24 h, 48 h,72 h, and 96 h, respectively, after injection.

##### Drug Treatment of AD Model Mice

APP/PS1 AD mice (*n* = 8 per group) were given daily nasal administration with 30 µL of HSA, and dcHGT NPs at an HSA dose of 10 mg kg^−1^ or donepezil (0.11 mg kg^−1^), clioquinol (0.14 mg kg^−1^) solution for 1 month, respectively. As controls, AD mice were given daily nasal administration with 30 µL of saline for 1 month.

##### MWM Behavioral Test

Morris water maze was used to test the spatial learning ability and spatial memory function of mice. The maze was made up with a metal circular pool (diameter: 100 cm; height: 50 cm) and an escape platform (diameter: 9 cm; height ≈30 cm). The maze was filled with 24–26 °C water, which was dyed white with acrylic paint. The water level remained ≈1 cm above in order to make sure the platform was submerged. Before the experiment, the escape platform was placed in the middle of a quadrant and held the position throughout the experiment. The four quadrants of the maze had corresponding visual cue symbols on the walls of the pool. In the first 5 days, the mice (*n* = 8 per group) received daily trials from three different quadrants. The latency experiment was limited to 60 s. If the mice fail to find the platform within the 60 s, it was guided to the platform and stayed for 15 s. The swimming path and escape latencies of mice were recorded by an image tracking system, and data were calculated with software (Ethovision XT, Panasonic, Japan). The spatial memory function of mice was measured on the sixth day. Briefly, the escape platform was removed and the mice were released to the maze to swim freely for 60 s. Spatial acuity was expressed as the percentage of time that the mice swam in the quadrant where the escape platform used to be located.

##### Acquisition and Analysis of Cortical EEG Signals in Mice

Cortical EEG signals of the right frontal and temporal cortex of mice were conducted using a medilog dynamic EEG recording system. The mice were fixed and anesthetized with intraperitoneally injected 10% chloral hydrate. Followed by disinfection, the scalp was cut lengthwise along the middle line between the ears and the eyes, and the skull was exposed. To fully expose bregma points, meninges were cleaned with hydrogen peroxide. Right frontal electrode site: 0.5 mm right to the middle line, 1.54 mm in front of the bregma points, and 3 mm deep inside the brain; right temporal electrode site: 1.75 mm right to the middle line, 2.06 mm behind the bregma points, and 1.4 mm deep inside the brain; reference electrode sites: 2 mm left to the middle line and 4 mm behind the bregma points. The ground electrode was located at the base of the tail. According to the above position, the skull was drilled hole and a needle electrode was inserted. After connecting the EEG recording system, cortical EEG signals were recorded within 15 min. Three segments of EEG signals with a stable baseline and few artifacts were selected for analysis, with a sampling frequency of 200 Hz, a sampling accuracy of 16 bt, and a sampling time of 5.12 s for each segment. Before the EEG power calculation, MatlabR2013b was performed to removed baseline and filtered EEGLAB. The total EEG power and the power ratio of each frequency band in the frontal and temporal regions of mice in each group were calculated by self‐programming, analyzed. and compared. Herein, the frequency band power ratio = the calculated frequency band power/total power × 100%.

##### Histological Observation of the Brain Tissues’ Coronal Sections

The mice were euthanized after the EEG signals were collected. The brain tissues were harvested and immediately put into the precooled electron microscope fixing solution. Followed by fixing in 2.5% glutaraldehyde fixation solution and 1% medilog osmium tetroxide fixation solution and ascending gradient ethanol dehydration, the brain tissues were embedded in Epon812 for further section. After cut into 80 nm ± thick slices, the slices were double‐stained by uranium acetate and lead citrate. Subsequently, the slices of brain tissues were observed by TEM (HITACHI‐7500) and photographed by a Megaview‐III digital electron microscope photography system. The slices were used for visualization of neurons and the morphology and number of synapses.

##### Histological Examination and Immuno‐Histochemical Staining

The brain tissue sections were stained with H&E following previous reports. The samples were subjected to observation under an optical microscope. H&E‐stained sections were visualized for morphological change of the cells of hippocampus in the brain. As for immuno‐histochemical staining, the brain tissue sections and cells were successively fixed, washed with PBS three times, and blocked with 0.5% Tritonx and 5% Bovine Serum Albumin (BSA) for 30 min at 37 °C. Followed by incubation of A*β*1‐42 antibody (1:1000), synaptophysin (1:1000), and GAP‐43 overnight at 4 °C, the samples then incubated by goat antirabbit immunoglobulin G (IgG) (H&L) FITC (1:1000) for 1 h at room temperature. After color developing and sealing, the sections were finally digitized using an Olympus fluorescence microscope (IX‐51).

##### Statistical Analyses

All data were expressed as mean ± standard deviation (SD). The significance was analyzed by one‐way analysis of variance (ANOVA) with Bonferroni tests (Morris water maze test) or unpaired Student's *t*‐test (two‐tailed) by using SPSS20.0 software. A *P*‐value of <0.05 was considered statistically significant.

## Conflict of Interest

The authors declare no conflict of interest.

## Supporting information

Supporting Information
