# Supplementary material for: A Novel Targeted and High‐Efficiency Nanosystem for Combinational Therapy for Alzheimer's Disease
Source: Adv Sci (Weinh). 2020 Aug 9;7(19):1902906. doi: 10.1002/advs.201902906 (PMC7539195; doi:10.1002/advs.201902906)
Supplement: Supplementary file 1 — Supporting Information [file ADVS-7-1902906-s001.pdf]

© 2020 Wiley-VCH GmbH

ADVANCED SCIENCE
Open Access

Supporting Information

for *Adv. Sci.*, DOI: 10.1002/advs.201902906

A Novel Targeted and High-Efficiency Nanosystem for Combinational Therapy for Alzheimer's Disease

Han Yang, Weihang Mu, Daohe Wei, Yue Zhang, Yue Duan, Jun-xiao Gao, Xiao-qun Gong, Han-jie Wang, Xiao-li Wu, Huaying Tao,* and Jin Chang**

Supporting Information

A Novel Targeted and High Efficiency Nanosystem for Combinational Therapy of Alzheimer's Disease

Han Yang[†,#], Weihang Mu[‡,#], Jun-xiao Gao[†], Daohe Wei[†], Yue Duan[†], Yue zhang[†], Xiao-qun Gong[†], Han-jie Wang[†], Xiao-li Wu[†*], Huaying Tao[§*], Jin Chang[†*]

† *School of Life Sciences, Tianjin University, 92 Weijin Road, Nankai District, Tianjin 300072, P.R. China*
‡ *Tianjin Chidren's Hospital, 238 Longyan Road, Beichen District, Tianjin 300072, P.R. China*
§ *Department of Nerology, Tianjin Medical University General Hospital, 154 Anshan Road, Heping District, Tianjin 300072, P.R. China*

* Corresponding author Xiao-li Wu, E-mail address: wuxiaoli@tju.edu.cn
Corresponding author Huaying tao, E-mail address:taohuaying@126.com
Corresponding author Jin Chang, E-mail address: jinchang@tju.edu.cn

# These authors contributed equally to this work.

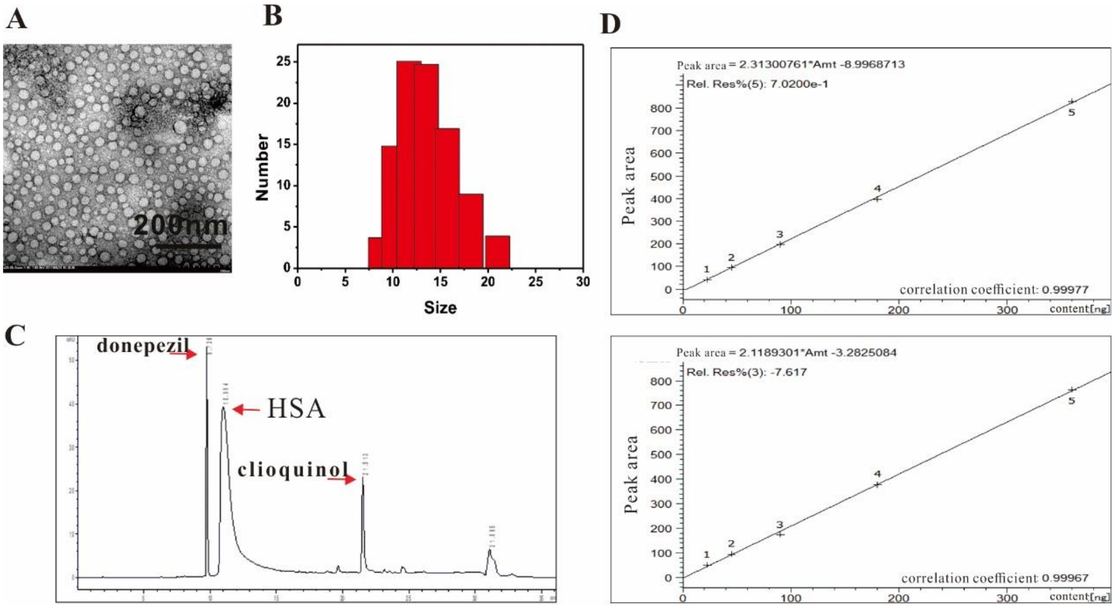

Figure S1. (A) TEM images of dcHG NPs (scale bar: 200 nm); and (B) DLS

measurement of dcHG NPs; (C) Absorption spectrum of dcHGT NPs measured by HPLC; (D) Standard curve of donepezil and clioquinol measured by HPLC.

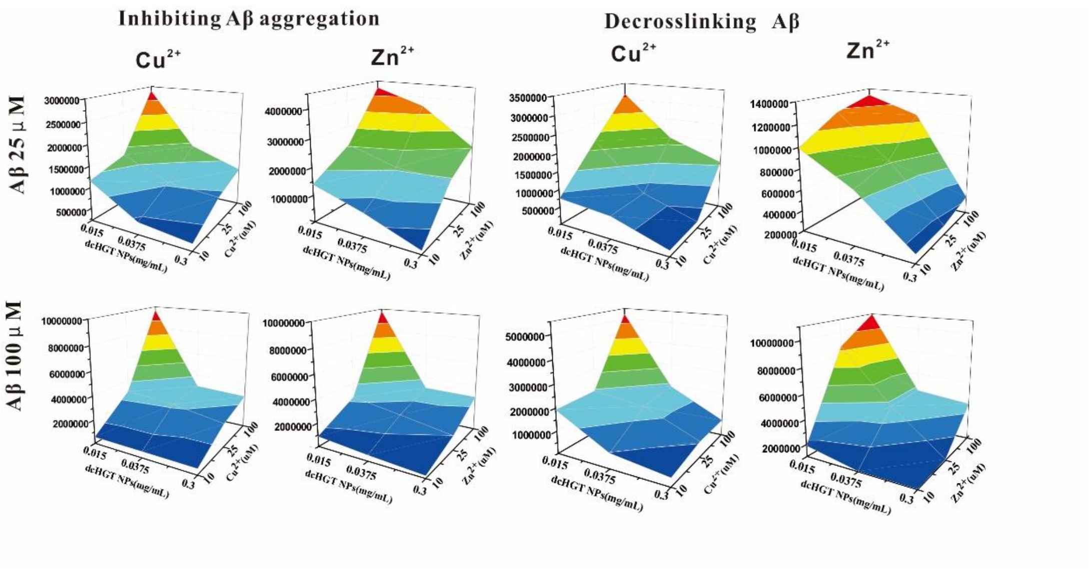

Figure S2. 3D color map surface of fluorescence signals in inhibition and disaggregation experiments ([Aβ] = 25,100 μM, [metal ions] = 10-100 μM, and [dcHGT NPs]=0.015-0.15mg/mL). Data are presented as the mean ± SD.

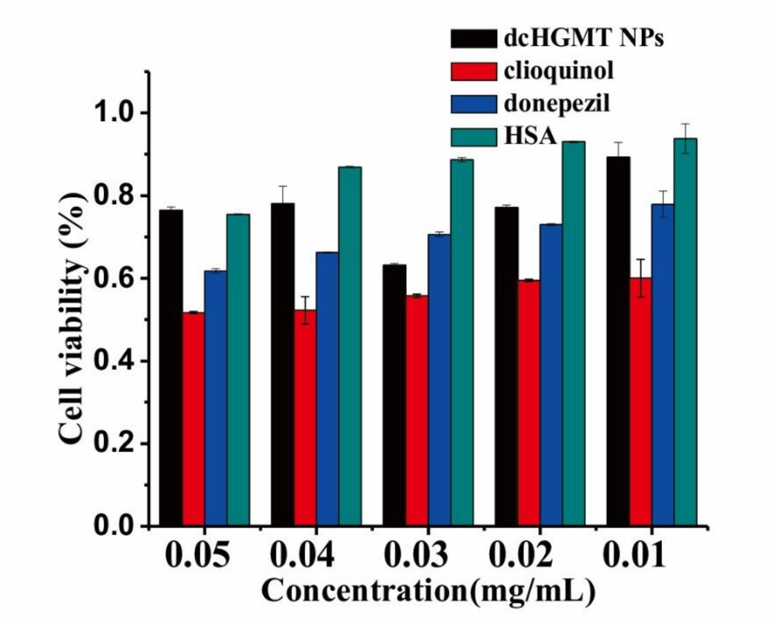

Figure S3. Cell viability of BV-2 cells incubated with free donepezil, clioquinol, HSA and dcHGT NPs at various concentrations. Data are presented as the mean ± SD.

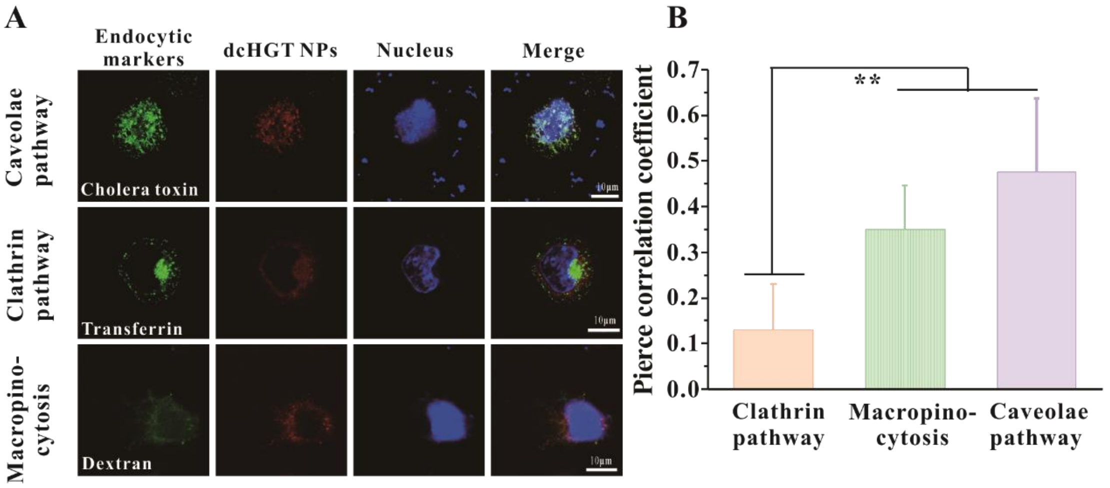

Figure S4. Cellular uptake of dcHGT NPs in BV2. (A) Representative images showing the co-localization of dye-labeled dcHGT NPs with endocytic markers by confocal microscopy. The nuclei were stained with Hoechst 33258 (blue). Scar bar, 10 µm. (B) Pearson's correlation coefficient analysis of the co-localization between dcHGT NPs and endocytosis markers in BV2. The quantitative data were obtained through Image J software. Data were expressed as mean ± SD (n = 6). ***P* < 0.01.

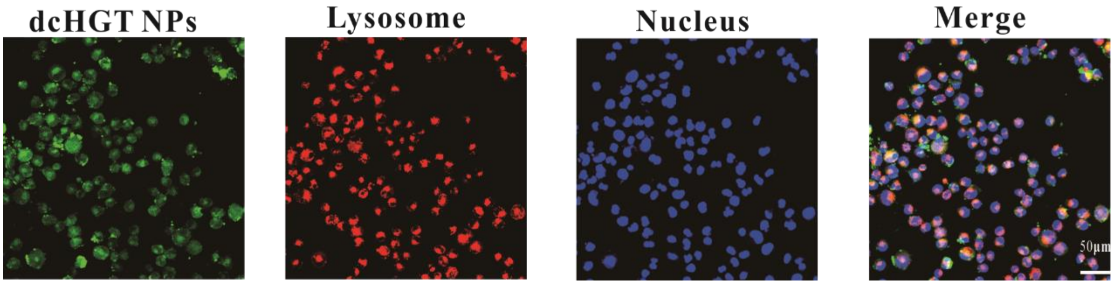

Figure S5. Colocalization (yellow) between dcHGT NPs (green) and LysoTracker (red), the indicator of lysosme, after 4 h incubation in BV2. Scar bar, 5 µm.

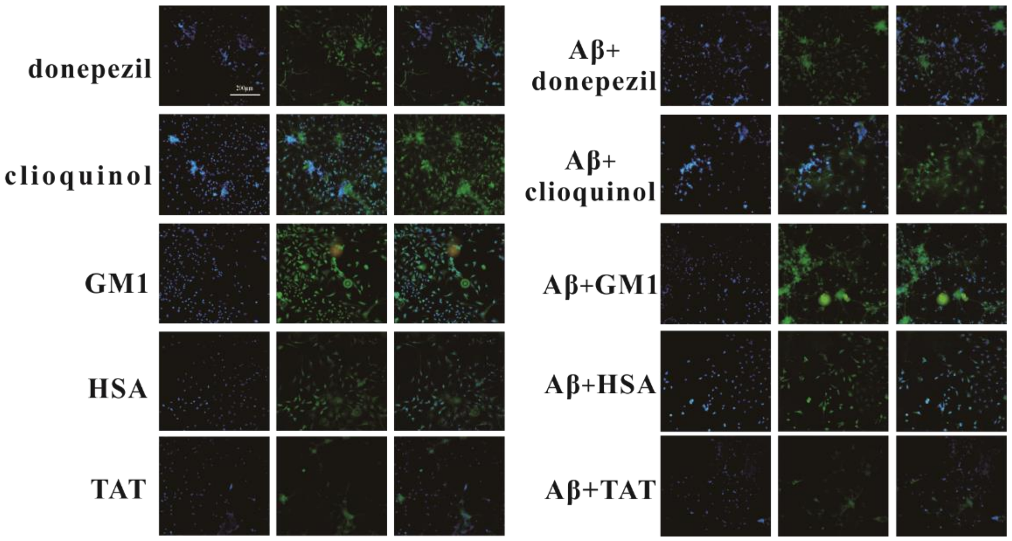

Figure S6. Fluorescent morphology images of the primary neurons captured by HCS; Scale bar: 200 μm.

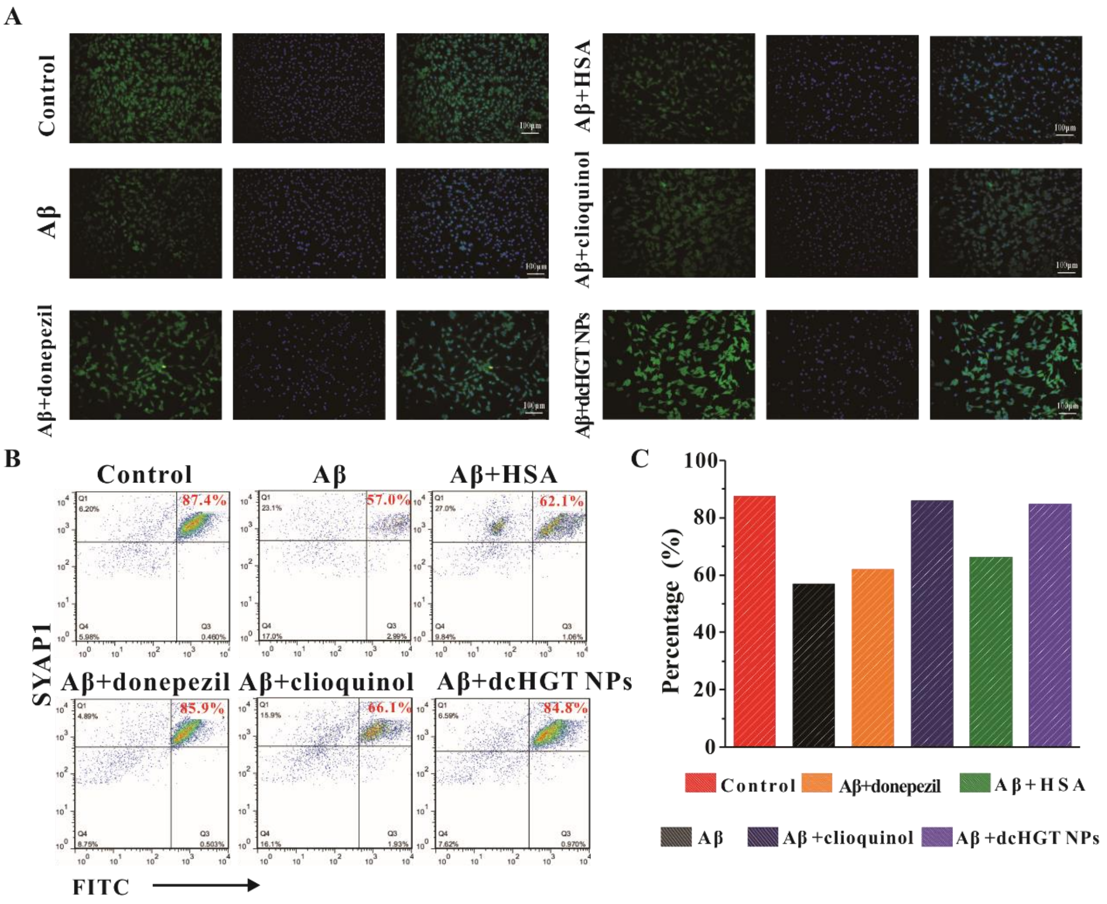

Figure S7. Immunofluorescence (A) and flow cytometry (B) were performed to

identificate the expression of synaptophsin (SYAP1) in PC12. (C) Statistical analysis of SYAP1 of PC12 in control, Aβ, Aβ+HSA, Aβ+donepezil, Aβ+clioquinol and Aβ+dcHGT NP groups.

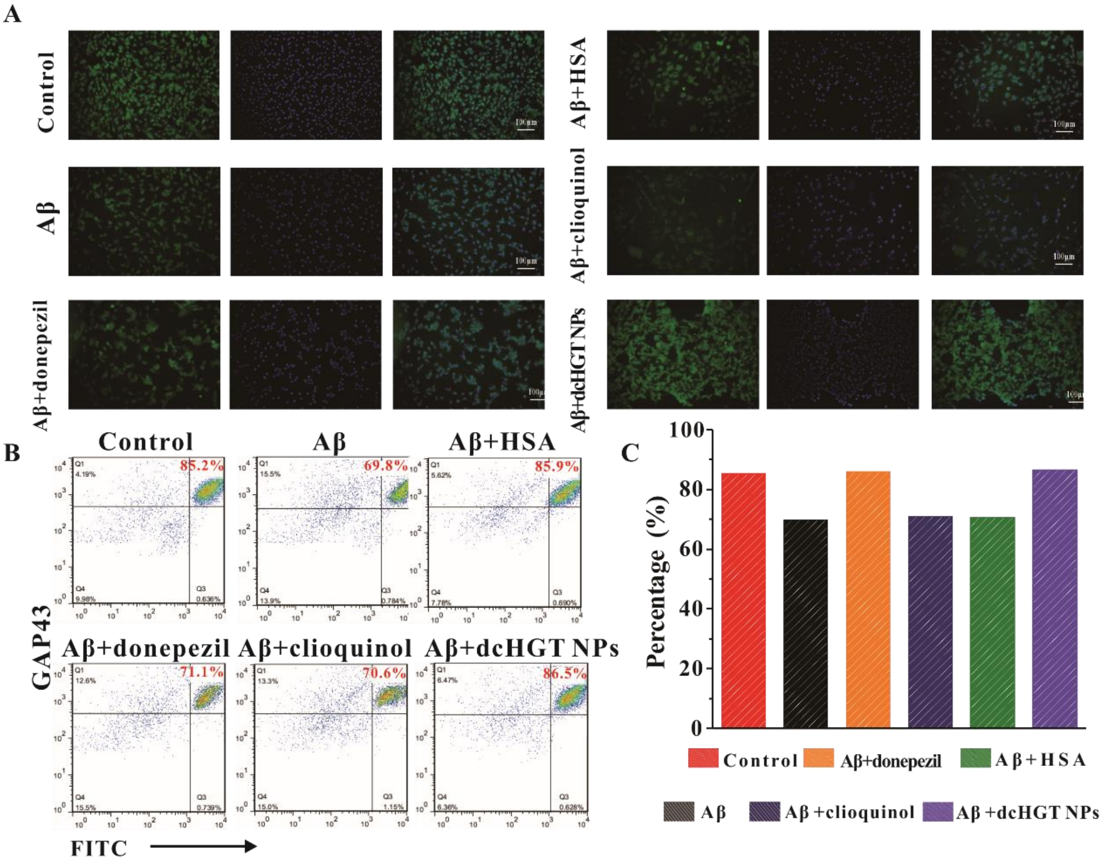

Figure S8. Immunofluorescence (A) and flow cytometry (B) were performed to identificate the expression of growth-related protein 43 (GAP43) in PC12. (C) Statistical analysis of GAP43 of PC12 in control, Aβ, Aβ+HSA, Aβ+donepezil, Aβ+clioquinol and Aβ+dcHGT NP groups.

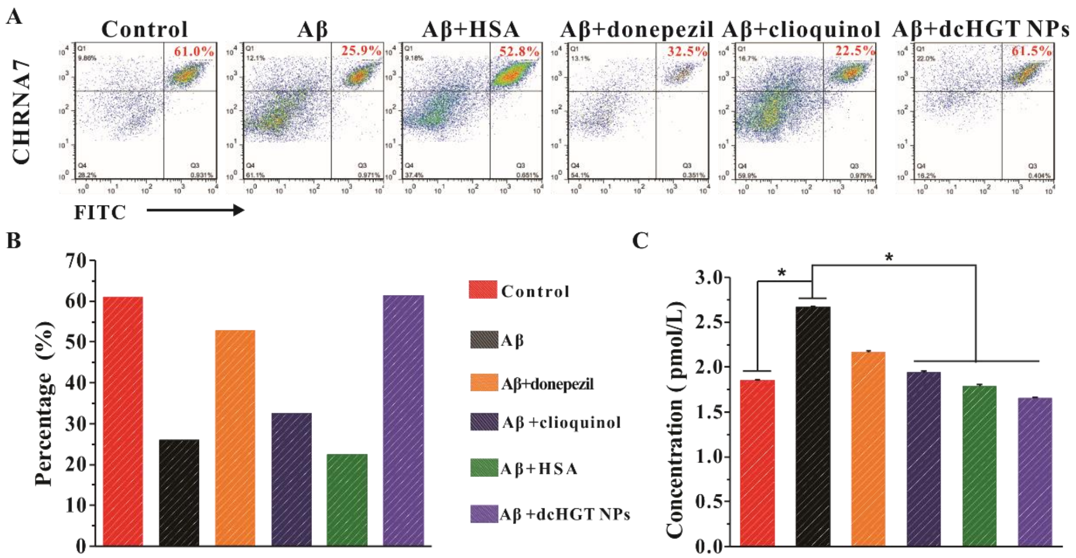

Figure S9. (A) Flow cytometry were performed to identificate the expression of neurotype 7 nicotine receptors (CHRNA7) in PC12. (B) Statistical analysis of CHRNA7 of PC12 in control, Aβ, Aβ+HSA, Aβ+donepezil, Aβ+clioquinol and Aβ+dcHGT NP groups. (C) Statistical analysis of the expression of caspase 3 in different groups by ELISA analysis. Data are presented as the mean ± SD. *$P < 0.05$.

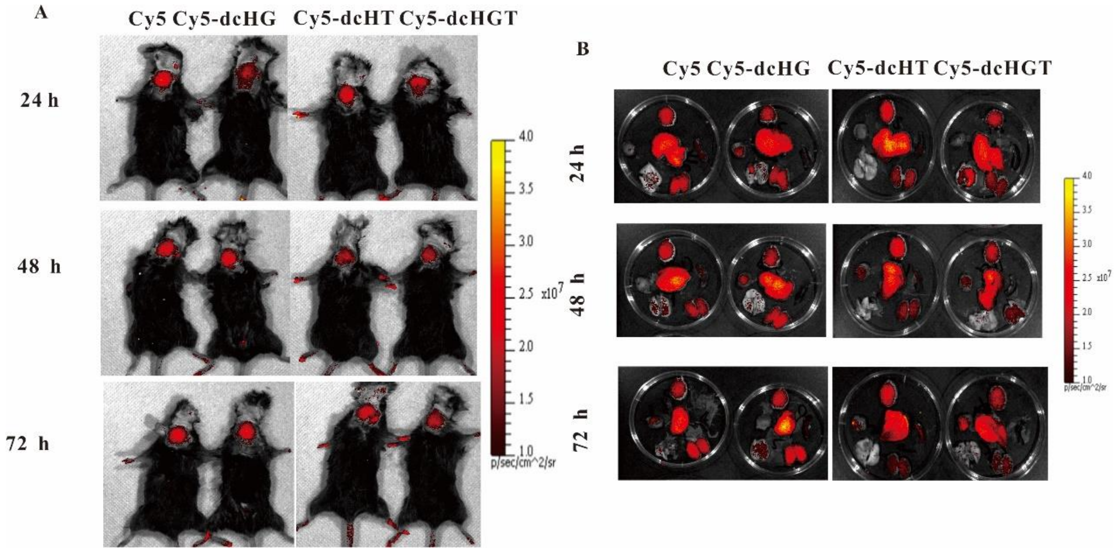

Figure S10. (A) In vivo fluorescence imaging at 24h, 48h and 72h after nasal administration of Cy5, Cy5- dcHG NPs, Cy5- dcHT NPs and Cy5- dcHGT NPs; (B) Ex vivo fluorescence images of brain and other tissues at 24h, 48h and 72h after

administration.

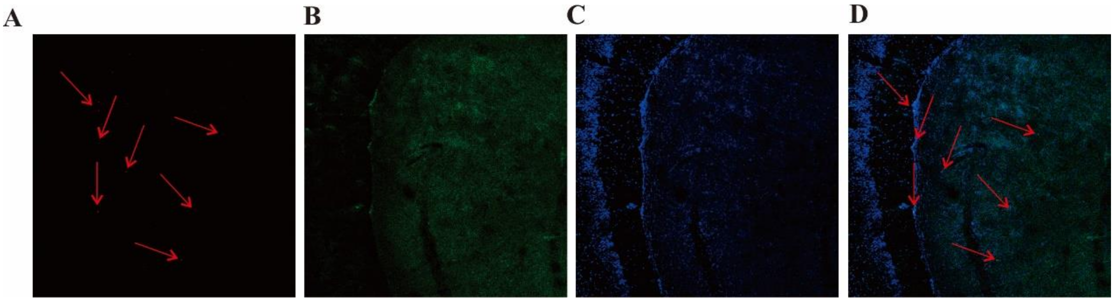

Figure S11. Fluorescence imaging of brain slices at 96h after nasal administration with Cy5-dcHGT NPs; (A) Cy5-dcHGT NPs, (B) immunofluorescent primary neurons, (C) DAPI, (D) merge images.
